# High-Frequency Ultrasound for Assessment of Peri-Implant Bone Thickness

**DOI:** 10.3390/jcm8101539

**Published:** 2019-09-25

**Authors:** Juliana Marotti, Sarah Neuhaus, Daniel Habor, Lauren Bohner, Stefan Heger, Klaus Radermacher, Stefan Wolfart

**Affiliations:** 1 Department of Prosthodontics and Biomaterials, Centre for Implantology, Medical School of the RWTH Aachen University, Pauwelsstrasse 30, 52074 Aachen, Germany; sarah.neuhaus@rwth-aachen.de (S.N.); lauren@usp.br (L.B.); swolfart@ukaachen.de (S.W.); 2 Department of Medical Engineering, RWTH Aachen University, Pauwelsstrasse 20, 52074 Aachen, Germany; daniel.habor@rwth-aachen.de (D.H.); radermacher@hia.rwth-aachen.de (K.R.); 3 Institute for Biomedical Engineering, Mannheim University, John-Deere-Strasse 85, 68163 Mannheim, Germany; s.heger@hs-mannheim.de

**Keywords:** ultrasonography, ultrasonic imaging, dental implant, bone–implant interface

## Abstract

Purpose: The aim of this study was to evaluate the accuracy of high-frequency ultrasound (HFUS) for measurement of bone thickness surrounding dental implants. Methods: Eight porcine bone samples containing dental implants were scanned by a HFUS scanner and compared using cone-beam computed tomography (CBCT) and an optical scanner. Bone thickness was measured in the buccolingual region of dental implants in 10 points distributed between the platform and apical portion of the implant. Results: The mean measurement error for the ultrasound method was 0.11 mm, whereas CBCT showed a measurement error of 0.20 mm. For both devices, the maximal measurement error was 0.28 mm. Conclusion: Within the simulated limited conditions of this study, high-frequency ultrasound, with optical scanning used as a reference, presented higher accuracy in comparison to CBCT, and seems to be a promising tool for measuring peri-implant bone.

## 1. Introduction

The success of a dental implant rehabilitation depends on the health of hard and soft peri-implant tissues [1]. In order to achieve an optimal functional and aesthetic result, total coverage of the dental implant by bone is required [2,3], since there is strong correlation between bone thickness and primary implant stability [4]. Furthermore, in cases in which the bone thickness is lower than 1 mm, gingival recession may occur and result in a negative aesthetic appearance [5]. 

After implant surgery, bone remodeling resulting from a biological process is expected, and studies have shown that the amount of vertical bone is variable considering biological and mechanical aspects [6,7,8]. However, in the case of serious complications, bone resorption may be advanced and result in loss of osseointegration and subsequent implant failure [9]. These slight changes in bone volume may be detected early by using diagnostic imaging to monitor the peri-implant bone—an important step in follow-up visits [10].

As cone-beam computed tomography (CBCT) is capable of providing cross-sectional images, it is considered the gold-standard method for imaging peri-implant bone, and may be useful for assessing the peri-implant tissues and implant situation after the procedure [1,11]. However, the occurrence of artifacts in the presence of dental implants may jeopardize visualization of the bone–implant interface and make it difficult to assess peri-implant bone [12,13]. Furthermore, CBCT is not recommended for evaluating asymptomatic implants in periodic exams due to exposure to high levels of radiation, and it should be used only in case of peri-implant bone defects or presence of symptoms [14,15]. These limitations may therefore hamper the detection of initial bone loss—data that would be useful to avoid the need for more invasive procedures and ensure the successful outcome of dental implants [16].

Thus, new technologies are required for the purpose of peri-implant bone assessment. Ultrasound has emerged in the field of dentistry and has shown reliable results for evaluating soft tissues [17,18], bone surfaces, and dental implants [1,19,20,21,22,23,24,25,26,27,28]. Furthermore, it is free of ionizing radiation and is capable of penetrating soft tissues [29,30], allowing the visualization of the dental implant and bone surface [19,23,26].

Recent studies have shown that high-frequency ultrasound (HFUS) is a reliable technique for assessing peri-implant bone [20,21,22,23]. In these studies, nominal frequencies in the range of 40 MHz were applied to meet the higher demands concerning resolution and accuracy. Ultrasound, at this frequency, however, is able to scan the bone surface through thin soft-tissue layers, but hardly its inner portion. To determine the exact three-dimensional (3D) position of a dental implant within the bone, a priori information from a dental implant containing a screw-retained crown (before placement in the bone) is necessary. For this purpose, an optical scanner is required. First, the implant with its crown is scanned with an optical scanner device. After the implant has been inserted into the bone surface, the ultrasound scan is performed. Considering that both the ultrasound and the optical scanner are capable of providing information about the crown surface, which is outside the bone, the two images can be matched. When this a priori information of a dental implant position has been obtained, the relationship between the dental implant and bone can be reconstructed, and the thickness of buccal bone surrounding the implant can be determined [20]. In a recent study [23] this technique was used in association with a lower-frequency ultrasound probe to determine the cortical bone thickness in a dental implant model, and the results showed values similar to those of the CBCT measurements. However, the accuracy of this technique must be further evaluated. Therefore, the aim of this study was to evaluate the accuracy of a HFUS for measuring peri-implant bone surrounding implants in comparison with CBCT and optical scanning as a reference method.

## 2. Materials and Methods 

### 2.1. Sample Acquisition

This study was conducted in accordance with national legislation on the use of animals for research. Since animal tissue obtained from a local butcher was used for this study, no ethical approval was required. In order to simulate the human jawbone, two porcine ribs were used in this study. Each sample was prepared by removing the soft tissue and cleaning the bone structure, in which four dental implants (Camlog Screw-line, Ø3.8 mm; L 11 mm, Camlog Biotechnologies, Wimsheim, Germany) were placed perpendicular to the bone surface. Each region of bone containing a dental implant was sectioned with a saw, totaling eight individual bone blocks. Afterwards, each bone block was fixed to an acrylic resin support (Palapress, Heraeus Kulzer, Hanau, Germany) and the set was attached to a brick block (LEGO System, Billund, Denmark). Thus, the position of samples was standardized to obtain a range of measurements. 

**Figure 1:**
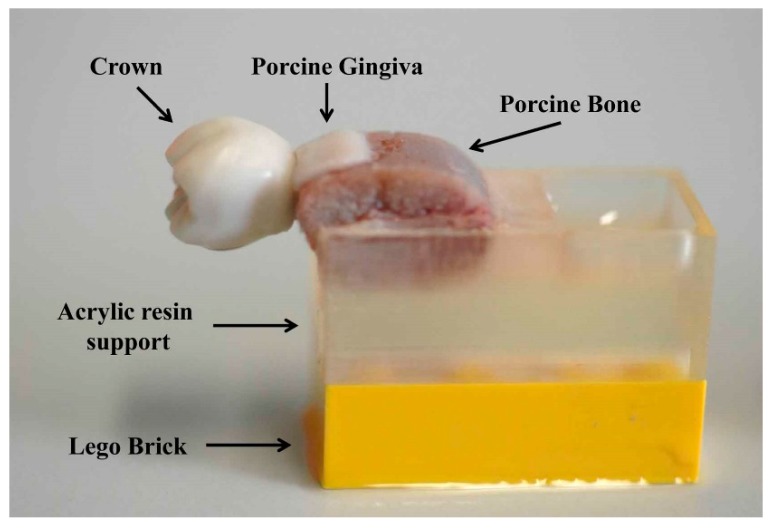
Bone sample with gingiva attached, a dental implant, and a superstructure.

For each implant, a crown was made by using an artificial resin tooth (Kavo Dental, Biberach, Germany), and inserting a screw into its inner part, allowing connection between the crown and dental implant by fastening the screw in a defined position. Samples of fresh porcine gingiva were attached to the vestibular portion of the bone blocks with glue (Loctite Super Kleber, Henkel AG & Co., Munich, Germany) applied only on the edges of bone, to prevent the glue from interfering in the acoustic coupling (Figure 1).

### 2.2. A Priori Information Acquisition

**Figure 2:**
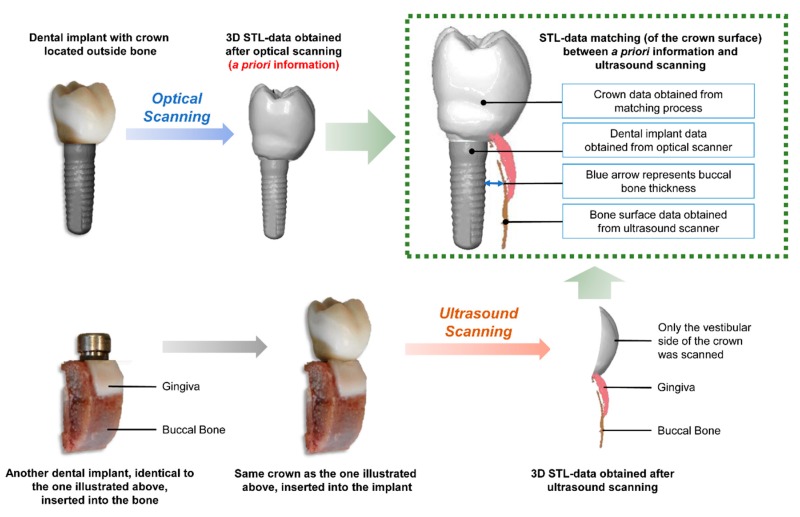
Priori information acquisition and 3D STL-data matching between optical and ultrasound scanning.

As HFUS hardly penetrate the inner portion of bone, a priori information from the dental implant and the crown was required to determine the implant axis relative to the superstructure (Figure 2). Thus, the dental implant and the respective crown were individually scanned by an extraoral scanner (D250, 3Shape, Copenhagen, Denmark). First, a powder was applied to the components, as recommended by the manufacturer. After this, the set was placed on a plate that rotated through 360°, allowing the entire piece to be scanned and creating a 3D-model with an accuracy lower than 20 µm. The position of the dental implant was determined by the GOM Inspect software (GOM GmbH, Braunschweig, Germany) based on a z-axis orientation.

### 2.3. Ultrasound

The ultrasound system set-up comprised a single element HFUS transducer (fc = 75 MHz; Aperture = 6.35 mm, f# = 2), a pulser-receiver (DPR500 Dual Pulser-Receiver, JSR Ultrasonics, Pittsford, USA) used in pulse-echo mode, and a 12-bit 400 MS/s analog-to-digital converter. The HFUS transducer was integrated into a mechanical set-up, allowing an object to be scanned in 4-axes, 3 translation and 1 rotation, with a mechanical precision lower than 10 µm. 

**Figure 3:**
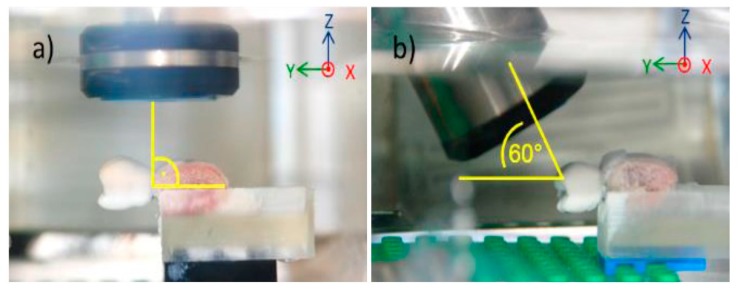
Scanning of porcine sample by means of ultrasound. Scanning was performed at an angle of 90° (**a**) and 60° (**b**) to the long axis of the dental implant.

**Figure 4:**
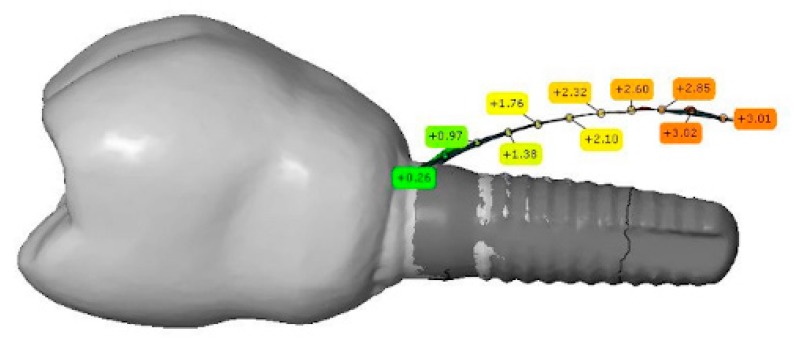
Matching between the a priori information and the ultrasound data. The numbers describe the thickness of bone above the implant surface. The color shades are defined as follows: orange corresponds to a thickness of 3 mm and green to a thickness of zero.

To propagate, ultrasound waves need a medium such as water. Thus, the scanning was performed by a HFUS prototype (developed by the Helmholtz Institute for Biomedical Engineering, RWTH Aachen University, Aachen, Germany) by immersing each sample in a water basin filled with tempered (37 °C) degassed isotonic saline solution (0.9% NaCl). Commercial multi-channel ultrasound array systems usually provide a compound scan mode. In this mode ultrasound scans acquired from multiple angles are merged together in order to enhance the homogeneity of the entire scan, especially if specular surfaces, like bone or dental crowns, are involved. However, those systems are built for standard clinical applications, and hence do neither provide the HFUS range nor the software flexibility required for this application. In order to optimize our HFUS scans with respect to the specular bone surface by use of a single-element system, each sample was scanned twice. During the first scanning, the ultrasound probe was positioned at an angle of 90° to the long-axis of dental implant, whereas in the second scanning the probe position was at an angle of 60° to the dental implant (Figure 3). For both scans, the grid spacing resolution was set to 50 × 50 µm and the depth of scanning was 4.4 mm. The resulting point clouds, which represented the surface of the superstructure, gingiva, and bone, were matched with the a priori information by means of the GOM Inspect software, according to Habor et al. [16]. In this case, the superstructure was used to determine the position of measurements (Figure 4). For the matching process, the surface points recorded with the ultrasound system were assigned to the superstructure. The point clouds of the ultrasound measurement and the 3D models from the extraoral scanner were further processed in GOM Inspect software by superimposing the respective surface model of the alveolar bone on the a priori information. The measuring points for which no measured value could be generated from the ultrasound point cloud, linear interpolation was carried out from the two nearest surface points.

### 2.4. CBCT

Images were acquired by means of a CBCT device (Sirona Galileos, Bensheim, Germany) and examined by two experienced examiners using the same software (Galileos Viewer, Bensheim, Germany). The images were captured with standardized parameters: 85 kV and 10 mA exposure, 30 s, and voxel 0.3 mm.

### 2.5. Optical Scanning

**Figure 5:**
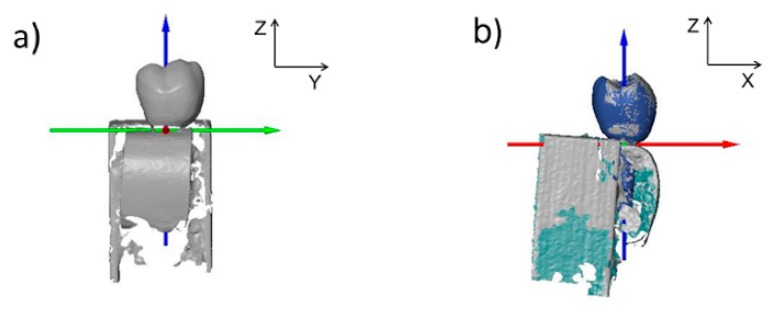
Acquisition of gold-standard values. Optical scan of porcine model (**a**) matched with the a priori information (**b**). The color shades at (**b**) are defined as follows: gray = outside surface; green = inside surface; and blue = a priori information.

Ultrasound based thickness measurements were compared with reference measurements taken on 3D models of the specimens acquired with the extraoral optical scanner previously mentioned (D250, 3Shape, Copenhagen, Denmark). The extra-oral optical scanner was used for reference measurements due to its capability of scanning the entire piece with a precision and accuracy of lower than 20 µm. Prior to the optical reference scan, the gingiva was removed from the specimens because the optical scanner was unable to detect the subgingival jawbone surface. A powder was applied on the bone surface as recommended by the manufacturer. The resultant three-dimensional surface models were matched to the a priori information to obtain the implant position (Figure 5).

### 2.6. Bone Thickness Measurement

**Figure 6:**
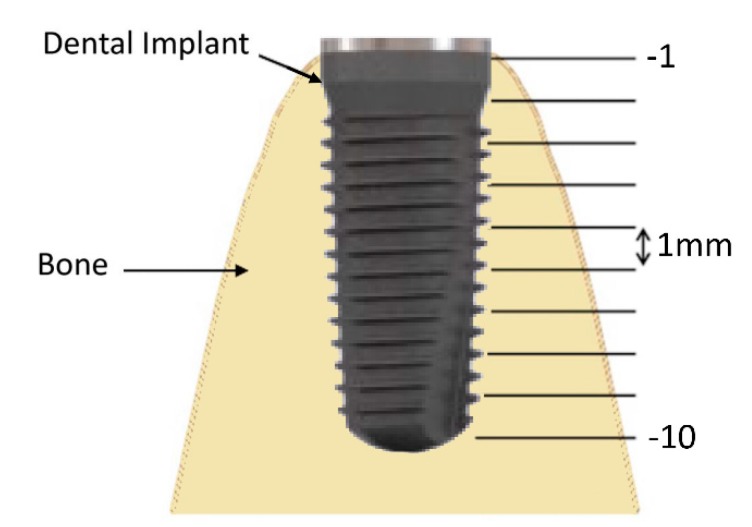
Points of measurement distributed along the bucco-lingual center of implant with a distance of 1 mm between two points.

In ultrasound scans, bone thickness was measured by one calibrated observer in 10 defined points. The reference for all measurements was the implant apex. The measurement depth of the implant apex was defined as −10 mm (minus sign here is related to the coordinate system). Points of measurement were distributed along the buccolingual center of the implant with a distance of 1 mm between two points (Figure 6). The implant apex could be identified in the CBCT scans, and thus the measurement points could be derived. In the HFUS data the implant shoulder could be identified. The implant length was well known, and thus the depth of the apex could be calculated. This depth was defined as −10 mm and measurements in the ultrasound data were taken relative to this depth in accordance with the CBCT measurements.

In CBCT images, the measurements were performed by two independent examiners using the Galileos Viewer software, as previously described. For ultrasound scans and optical scan values, the software GOM Inspect was used to perform the measurements.

### 2.7. Data Analysis

Mean, standard deviation and maximum values of bone thickness were determined for each group. First, each group was analyzed separately. Thus, measurements obtained by ultrasound at two different angles were compared. Afterwards, the images were superimposed on the optical scan image and a deviation analysis was performed. Measurements of CBCT images obtained by two examiners were analyzed by determining the mean difference between them.

Secondly, measurement values obtained by the three groups were manually compared. The measurement error of ultrasound and CBCT in comparison with optical scan was determined. For ultrasound, the values were described as ∆US (HFUS—optical scanner measurements). The CBCT values were described as ∆CBCT (CBCT—optical scanner measurements).

## 3. Results

The average deviation between the ultrasound point cloud and the a priori optical surface model, which resulted in the best-fit orientation, was about 26 μm. In this case, there were approximately 40,000 points per superstructure. 

**Figure 7:**
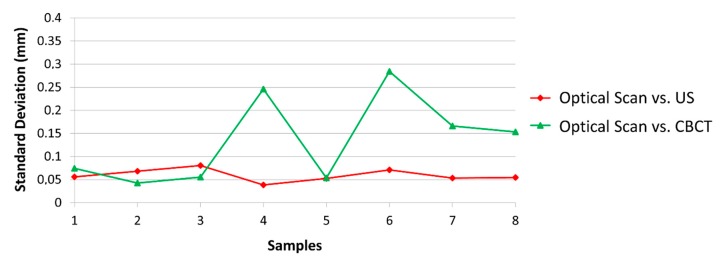
Standard deviation of all samples in relation to the optical scan. CBCT: cone-beam computed tomography; US: ultrasound.

Figure 7 shows the standard deviation of all measurements and samples in relation to the optical scan. The highest SD value of the HFUS measurement was 0.08 mm, whereas for CBCT it was 0.28 mm.

**Figure 8:**
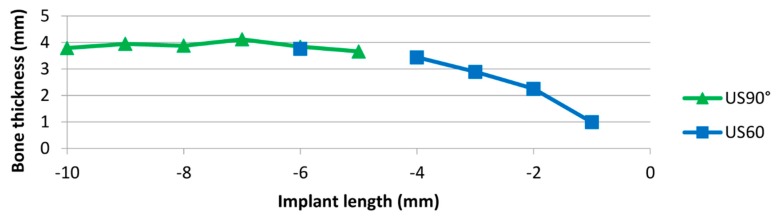
Bone thickness vs. implant depth for ultrasound at 60° and 90°.

**Figure 9:**
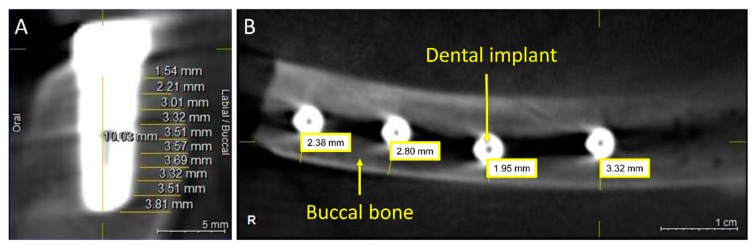
CBCT image of a dental implant and measurement points in the sagittal (**A**) and horizontal (**B**) view.

With regard to ultrasound, the scanning at 90° provided a better result for the apical third, whereas the scanning at 60° showed a better delimitation of borders for the cervical third (Figure 8). For deviation analysis, the 90° scanning showed a mean value ranging between 0 and 0.13 mm, with the highest deviation of 0.34 mm. For the 60° scanning the mean values ranged between 0.04 and 0.11 mm, and the highest value was 0.90 mm. For CBCT images (Figure 9), the mean difference of measurement performed by two examiners was 0.42 mm. 

With regard to bone thickness, the optical scanner, CBCT and ultrasound showed similar measurement values at some points. When analyzing the measurement error obtained from ultrasound and CBCT in comparison with the extra-oral scanner, the mean value of ΔUS was 0.11 mm, with a maximum value of 0.28 mm and the highest standard deviation of 0.08 mm. For ΔCBCT, the mean value of measurement error was 0.2 mm, with a maximum value from 0.64 mm and the highest standard-deviation of 0.28 mm.

## 4. Discussion

Ultrasound showed a higher accuracy in comparison with CBCT, while its measurement error was closer to the optical scan values to a small degree. Furthermore, the standard deviation shown by ultrasound was lower than that of CBCT. According to Tapie et al. [31], accuracy is determined by the closeness to the true value. Hence, it consists of not only the trueness of the result, but also of agreement among the different measures. In this case, ultrasound could be suggested as a promising technique for bone surface determination.

Considering that the bone shape where the dental implant is placed is not straight, but convex, inaccuracies can occur if the ultrasound scan only hits the bone from one direction. Thus, to avoid this approach, measurements were performed at 60° and 90°. In Figure 6, the reason why the data points for the scan at 90° showed more apical points, while the scan at 60° scan showed more coronal points was because ultrasound provides larger echo amplitudes when the wave hits the bone in or closer to a vertical direction. In the coronal area, the 60° direction was better aligned to the bone surface.

The use of ultrasound for dental scanning, maxillofacial fractures, diagnosis of peri-implant bone loss, among other purposes, has been reported in the literature [21,23,24,26,32,33,34,35,36]. Choi et al. [19] evaluated the use of 2D ultrasound to image soft and hard tissues of interest for dental implantology. A 24-MHz frequency ultrasound system was used to scan porcine jaws, in which dental implants were inserted with and without the presence of bone dehiscence. According to the authors, the bone and implants below the soft tissue were clearly visible and ultrasound had the potential to be used for determining bone surface morphology. However, a limitation of the technique has been reported with regard to penetration into the inner bone, and visualization of the dental implant in contact with this bone. Bertram and Emshoff [10] showed an underestimation of bone loss measured by ultrasound in 25 patients. Nevertheless, the technique was considered reliable for evaluating peri-implant bone loss. With regard to the HFUS, use of the ultrasonic system described in this study has previously shown accuracy for bone thickness determination [20] comparable with that of CBCT [23]. 

It must be emphasized that the accuracy of CBCT depends on the exposure parameters. Studies have shown that the voxel size plays an important role in determining the visualization of anatomical structures [11,13,28,37]. In case of dental implants, artefacts can be found, which may hamper a clear visualization and precise bone measurements. The fact that the authors did not opt to use a software to optimize the image quality should be pointed-out as a limitation of this study.

The main significance of this study is to present a new technology for evaluating peri-implant bone. Although tomographic techniques have been widely used to determine bone loss [38,39,40,41], in presence of dental implants CBCT has shown some drawbacks, such as the exposure of patients to ionizing radiation and presence of metal artifacts, which may make it difficult to visualize the bone–implant surface and jeopardize the measurements performed by clinicians [13]. This may lead to underestimating the bone thickness, as described in previous studies [13,28]. 

In view of the potential events of biological bone remodeling and vertical peri-implant bone resorption [42], the detection of bone thickness with measurement error below 0.2 mm may suggest that it would be advantageous to use ultrasound (in comparison with CBCT), considering that it would be able to detect not only biological changes, but also to provide an early diagnosis of peri-implant bone loss. This is in agreement with previous studies showing that US was accurate measuring peri-implant bone defects when compared to CBCT [43,44]. 

Some differences in image acquisition between CBCT and ultrasound are pointed out. First of all, the principle of ultrasound is the reflection of ultrasonic waves when these are in contact with the interface of two media with acoustic impedance. Hence, in biological tissues with a high capacity of reflection, such as bone, less ultrasound energy is able to penetrate the inner portion of the bony tissue. On the other hand, radiographic images involve the emission of x-rays, which are absorbed by tissues according to their density and allows evaluation of the inner portions of tissues [19]. In this study, an important step in the methodology used was the acquisition of a priori information (acquired with an optical extraoral scanner and used for the reference scans), which helped to determine the three-dimensional relationship between the bone and implant. However, this information can also be acquired with the ultrasound system presented in this study. A conceivable solution for the future could be a scan body or 3D-models of the implants provided by each manufacturer in order to obtain this a priori information and perform the scans. 

The extraoral optical scan was used for reference scans due to its ability to provide a 3D model with high accuracy. Nevertheless, systematic geometric defects due to the digitization process [31] must be considered a limitation of this in vitro study. Furthermore, the complexity of the oral environment plays an important role in diagnostic imaging, and further studies are required to determine the feasibility of this technique for clinical use.

## 5. Conclusions

Within the simulated limited conditions of this study, high-frequency ultrasound, with optical scanning used as a reference, presented higher accuracy in comparison to CBCT, and seems to be a promising tool for measuring peri-implant bone.
